# Comparison of Genotype Imputation for SNP Array and Low-Coverage Whole-Genome Sequencing Data

**DOI:** 10.3389/fgene.2021.704118

**Published:** 2022-01-03

**Authors:** Tianyu Deng, Pengfei Zhang, Dorian Garrick, Huijiang Gao, Lixian Wang, Fuping Zhao

**Affiliations:** ^1^ Institute of Animal Science, Chinese Academy of Agricultural Sciences, Beijing, China; ^2^ A. L. Rae Centre of Genetics and Breeding, Massey University, Hamilton, New Zealand

**Keywords:** genotype imputation, SNP density, reference population size, imputation accuracy, SNP chip, sequencing

## Abstract

Genotype imputation is the term used to describe the process of inferring unobserved genotypes in a sample of individuals. It is a key step prior to a genome-wide association study (GWAS) or genomic prediction. The imputation accuracy will directly influence the results from subsequent analyses. In this simulation-based study, we investigate the accuracy of genotype imputation in relation to some factors characterizing SNP chip or low-coverage whole-genome sequencing (LCWGS) data. The factors included the imputation reference population size, the proportion of target markers /SNP density, the genetic relationship (distance) between the target population and the reference population, and the imputation method. Simulations of genotypes were based on coalescence theory accounting for the demographic history of pigs. A population of simulated founders diverged to produce four separate but related populations of descendants. The genomic data of 20,000 individuals were simulated for a 10-Mb chromosome fragment. Our results showed that the proportion of target markers or SNP density was the most critical factor affecting imputation accuracy under all imputation situations. Compared with Minimac4, Beagle5.1 reproduced higher-accuracy imputed data in most cases, more notably when imputing from the LCWGS data. Compared with SNP chip data, LCWGS provided more accurate genotype imputation. Our findings provided a relatively comprehensive insight into the accuracy of genotype imputation in a realistic population of domestic animals.

## Introduction

The availability of next-generation sequencing technologies has made it possible to take account of whole-genome sequencing (WGS) data for genome-wide association studies (GWASs) or genomic prediction (GP) (Koboldt et al., 2013; Ni et al., 2017). However, whole genome resequencing is typically more expensive than SNP chip genotyping in most species, precluding deep sequencing of every individual in a population. Accordingly, over the past decade, the application of GWAS and GP has mainly been based on SNP chip data. The content of SNP arrays have typically been chosen from a database comprising relatively small numbers of sequenced individuals, which can result in ascertain bias (Lachance and Tishkoff, 2013). Although SNP chips tend to be cost-effective compared to sequencing, they cannot capitalize on all the genomic information if the SNPs on the chip array have incomplete linkage disequilibrium with the causal mutations. Furthermore, they do not provide the understanding of the causal mutation that can be obtained by annotation of highly significant sequence variants. One option is to impute SNP array genotypes to sequence resolution based on a reference population of a small number of deeply sequenced relatives. Another option is imputation from a large number of sparsely sequenced individuals, obtained from low-coverage whole-genome sequencing (LCWGS). Compared to SNP chip data, LCWGS can expose the segregating sequence variants and mitigate the ascertainment bias from SNP array.

Regardless of whether SNP arrays or LCWGS are used to characterize genotypes, imputation is an essential step in a GWAS or as a precursor to genomic prediction (Li et al., 2009; Al Kalaldeh et al., 2019). Imputation can infer unobserved genotypes in a sample of individuals that have higher genotyping density from an SNP array, LCWGS, or WGS. Since WGS data should contain all genomic variants including causal mutations, it can increase the probability that causal variants can be directly identified. Accordingly, imputation can boost the power of GWAS analyses, improve the accuracy of GEBV in genomic prediction, be the basis for fine mapping, and facilitate meta-analysis that combines multiple studies based on different types of marker sets (Druet et al., 2014; Al-Tassan et al., 2015; Song et al., 2019).

Orho-Melander et al. (2008) imputed untyped HapMap SNPs to carry out fine-mapping and consequently found that GCKR rs780094 was associated with opposite effects on fasting plasma triglyceride concentrations. Many novel loci that increased the risk of type 2 diabetes were identified using high-density imputation (Mahajan et al., 2018). Association statistics obtained using imputed data from ultra low-coverage (0.24x) sequencing data attained similar *p*-values at known associated variants to those which had been obtained using an SNP chip (Pasaniuc et al., 2012). Huang et al. (2015) used imputation to construct a genome map for 1,495 elite hybrid rice varieties and their inbred parental lines and investigated 38 agronomic traits. They identified 130 associated loci which proved that the accumulation of numerous rare superior alleles with positive dominance was an important contributor to the heterotic phenomena.

The advent of low-cost next-generation sequencing has led to a rapid increase in the size of publicly available reference data sets. For example, the 1,000 Bull Genomes Project (http://www.1000bullgenomes.com/) has now sequenced thousands of animals and obtained about 155 million genetic variants representing many of the world’s cattle breeds, providing a high-quality reference population (Georges, 2014; Hayes and Daetwyler, 2019). Many studies have used the variants in that reference population for imputation to new datasets to improve the accuracy of genomic prediction or to identify new candidate genes (Ibeagha-Awemu et al., 2016; Aliloo et al., 2018).

However, using low-quality imputed data may not lead to reliable GWAS or higher accuracy in genomic predictions (van Binsbergen et al., 2014). Multiple factors can affect the imputation accuracy, including size of the imputation reference panel, the imputation method, the minor allele frequency of the variant being imputed, the accuracy of phasing that constructs haplotypes in the reference and the study samples, and the sequencing coverage of the reference panel (Das et al., 2018). Although some of the effects of these factors have been analyzed separately, a comprehensive analysis that jointly considered these factors would help users design more powerful datasets for GWAS or genomic prediction.

## Methods and Materials

### Simulation

**FIGURE 1:**
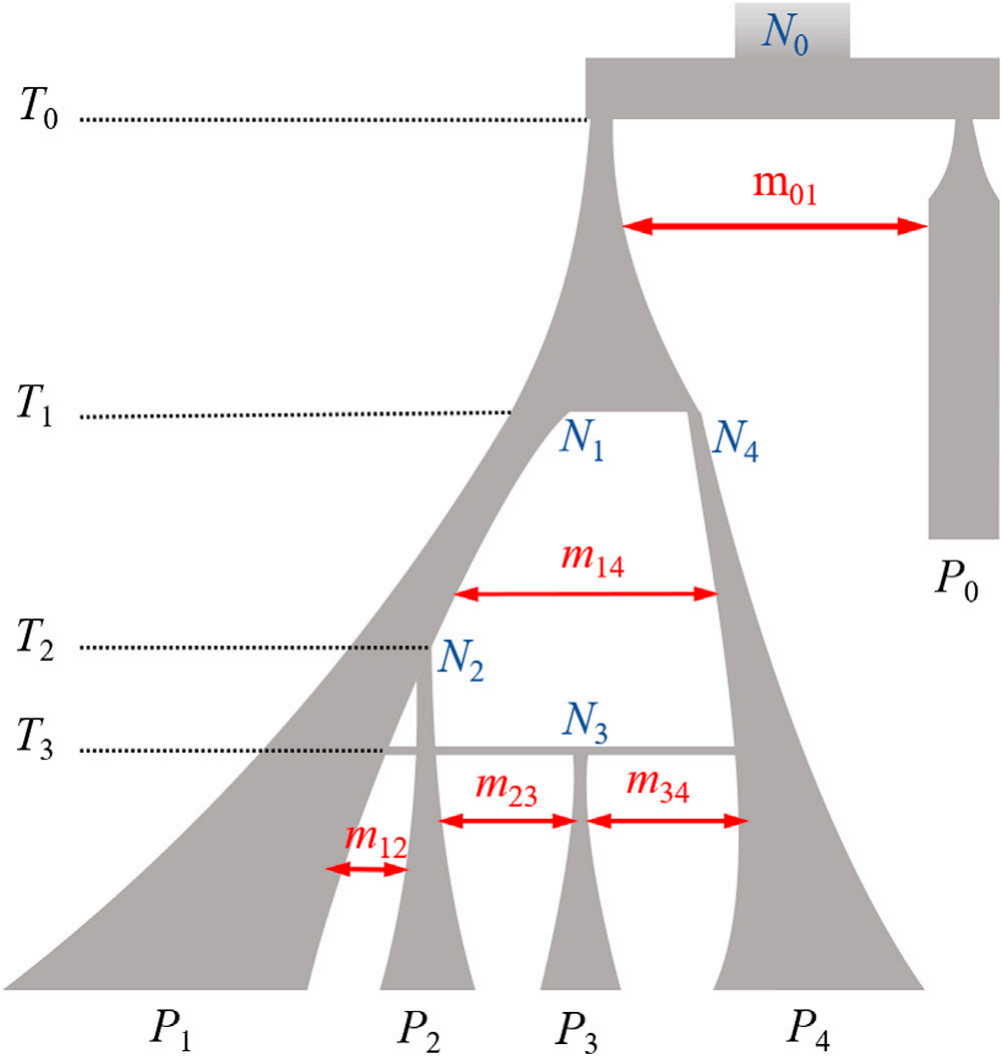
Illustration of the simulation demographic model. Branch width corresponding to population size and time flowing from the top to the bottom. The width of the double arrows is proportional to the migration intensity. *T*_i_, number of generations back to the population history event; *N*_j_, effective population size of *P*_j_ population; *m*_xy_, migration rate between *P*_x_ and *P*_y_. Model details are provided in Table 1 and Supplementary Text S1.

In this study, we employed simulations based on coalescence theory using *msprime* software to simulate sequence resolution data that are compatible with our knowledge of the demographic history of pigs (Pérez-Enciso, 2014). Pig populations experienced genetic mutation, migration, and bottleneck effects (Giuffra et al., 2000; Kim et al., 2002; Frantz et al., 2015), and the detailed parameters used are shown in Table 1. Following 58,000 simulated generations, four separate but related populations were simulated, which we refer to as *P*_1_, *P*_2_, *P*_3_, or *P*_4_ according to their genetic distance (Figure 1). In these four populations, there were a total of 20,000 diploid samples with 10 Mb of simulated sequence data. The *P*_1_ population included 11,000 individuals, while each of the other three populations had 3,000 samples. The first 1,000 individuals from *P*_1_ represented the target population for imputation. We randomly selected biallelic variants with MAF ≥ 0.01 in the target population to generate LCWGS data, and then selected evenly spaced markers at various densities to represent SNP chip data.

**TABLE 1:** Parameters used of the simulation with *msprime*.

Population history structural factors	Parameters				
Chromosome length			10,000 000 bp (10 Mb)		
Mutation rate			1 × 10^−7^		
Recombination rate			1 × 10^−7^		
Number of generations back to the population history event	*T*_ori_ = 58,000	*T*_0_ = 9,000	*T*_1_ = 3,000	*T*_2_ = 200	*T*_3_ = 20
Migration rate	*m*_01_ = 2.1 × 10^−5^	*m*_12_ = 1.1 × 10^−3^	*m*_14_ = 3.7 × 10^−4^	*m*_23_ = 5.2 × 10^−5^	*m*_34_ = 1.6 × 10^−3^
Effective population size	*N*_0_ = 10,873	*N*_1_ = 1,600	*N*_2_ = 1,200	*N*_3_ = 1,000	*N*_4_ = 1,400

We used the WGS data to calculate the average kinship coefficients in a pair-wise fashion between individuals in these populations, as in Table 2. The kinship coefficients between *P*_1_ and *P*_2_–*P*_4_ decrease successively, reflecting the increases in the genetic distances separating them.

**TABLE 2:** Genetic relationship between pair-wise populations.

Population	Average kinship coefficient		
	*P*_1_	*P*_2_	*P*_3_
*P*_2_	0.0070 (−0.065∼0.522)^a^		
*P*_3_	0.0027 (−0.077∼0.394)	0.0030 (−0.070∼0.510)	
*P*_4_	0.0011 (−0.083∼0.217)	0.0013 (−0.080∼0.270)	0.0184 (−0.059∼0.519)

a Range of kinship coefficients, with minimum to maximum in parentheses.

### Factors Influencing Imputation Accuracy

We took four factors affecting imputation accuracy for LCWGS and SNP chip data into account. These were the proportion of SNP markers relative to target sequence variants (i.e., SNP chip density), imputation reference population size, genetic distance between target and imputation reference population, and the methods of imputation. Table 3 lists the levels of each factor considered. A total of 336 scenarios representing all the factorial combinations of these levels were analyzed. In terms of SNP density, we set six levels where 1, 5, 10, 30, 50, or 90% of genomic biallelic variants were present on the SNP chip or LCWGS, the target marker number or density in reference populations are shown in Table 4. In the *P*_1_ population, we selected 100, 1 k, 3 k, 5 k, or 10 k simulated individuals to represent the imputation reference population but did not include any of the target individuals. For each of the other three populations, we set three levels of 100, 1 k, and 3 k of imputation reference samples.

**TABLE 3:** Levels of each factor to define the imputation scenarios.

Factors	Levels					
Reference population size	100	1,000	3,000	5,000	10,000	
Proportion of target markers/SNP density	1%	5%	10%	30%	50%	90%
Reference population	*P*_1_	*P*_2_	*P*_3_	*P*_4_		
Imputation method	Beagle5.1		Minimac4			
Data type	Chip data		Sequencing data			

**TABLE 4:** Number of segregating genetic variants in four simulated populations.

Proportion of target markers^a^	Reference population				Marker density (SNPs/kb)
	*P*_1_	*P*_2_	*P*_3_	*P*_4_	
Total^b^	212,696	214,899	216,366	213,389	21.4
1%	2,126	2,148	2,163	2,133	0.2
5%	10,634	10,744	10,818	10,669	1.1
10%	21,269	21,489	21,636	21,338	2.1
30%	63,808	64,469	64,909	64,016	6.4
50%	106,348	107,449	108,183	106,694	10.7
90%	191,426	193,409	194,729	192,050	19.3

a Represents the relative density of the pre-imputation marker panel.

b Total reflects the number of sequence variants targeted for imputation.

Imputation for every scenario was undertaken using Beagle5.1 (20Nov19.573) in comparison to Minimac4 v1.0.0, both with default parameter settings. Each program was run using its specific formats for reference panel data (bref3 for Beagle5.1 and m3vcf for Minimac4). We used Minimac3 to construct the m3vcf files. All imputation analyses were run on a dedicated 24-core 2.1-GHz workstation with an Intel Xeon Silver 4116 CPU and 128 GB of memory, and we evaluated one program at a time using five computational threads.

### Assessment of Imputation Accuracy

Imputation reliability and the error rate were used as the two criteria to assess imputation accuracy. The imputation reliability is the squared Pearson correlation coefficient between the imputed genotypes and the true genotypes at a specific locus. The genotypes were coded as 0, 1, or 2, corresponding to the homozygous reference allele, heterozygous alternative allele, or homozygous alternative allele. The equation can be written as follows:

$$r_{i}^{2}=\frac{{\left(Cov\left(X_{i},Y_{i}\right)\right)}^{2}}{Var\left(X_{i}\right)Var\left(Y_{i}\right)}$$

where $r_{i}^{2}$ is the imputation reliability for locus *i*; $X_{i}$ is a vector of the imputed genotypes at locus *i* and $Y_{i}$ is a vector of the true genotypes of imputed individuals at locus *i*.

The error rate refers to the percentage of loci that have wrongly imputed alleles:

$$er\left(\%\right)=\frac{n_{imputed\neq true}}{n_{imputed}}\times 100$$

where er(%) = the allelic imputation error rate, $n_{imputed\neq true}$ is the number of imputed alleles not equal to the true alleles, and $n_{imputed}$ is the number of alleles imputed.

We allocated the markers into several bins according to their MAFs and reported the average values of the imputation reliability and the error rate for all the markers within each bin. Furthermore, we calculated the regression of the imputation reliability or the error rate on the levels of each factor to determine if the factor had a significant effect (*p* < 0.05). We also report the correlation between the levels of each factor with the imputation reliability or the error rate. We used coefficients of variation (CVs) of the imputation reliability and the error rate to characterize imputation accuracy. The imputation computing time taken is reported for each scenario.

## Results

### Factors Affecting Imputation Reliability

**FIGURE 2:**
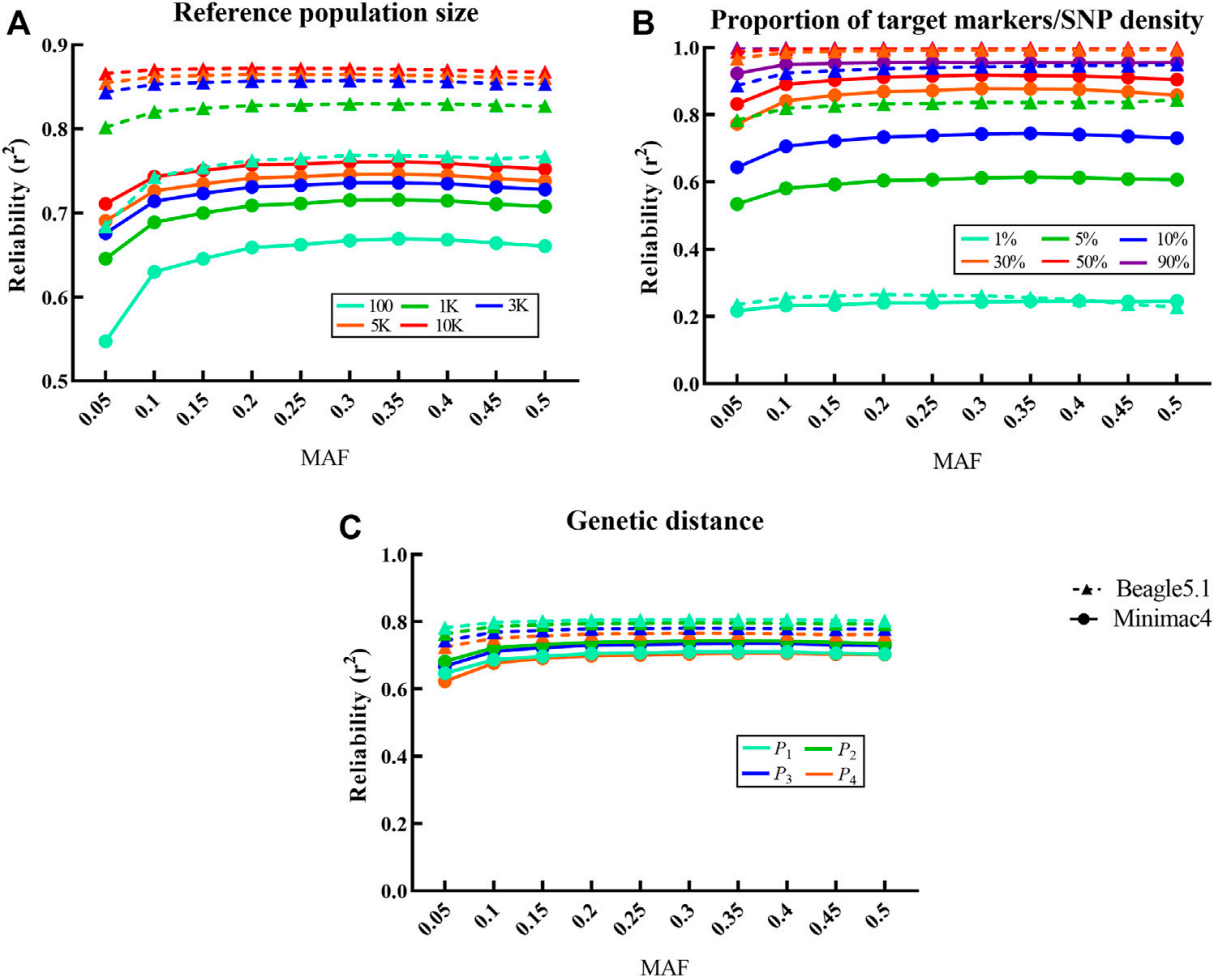
Influence of different factors on imputation reliability in LCWGS data. For each fixed level of the factors under each scenarios, the average at different levels of all other factors is taken as the reliability. Imputed alleles are binned according to their MAF count in each scenarios. Dotted line with a triangle sign represents Beagle5.1, while the solid line with a round sign represents Minimac4. Different colored signs represent different levels. **(A)** Influence of reference population size on imputation reliability. **(B)** Influence of the proportion of target markers or SNP density on imputation reliability. **(C)** Influence of genetic distance between reference population and target population on imputation reliability.

Significant differences in imputation reliabilities when imputing the sequence data were observed with regard to reference population size. Beagle5.1 typically outperformed Minimac4 with regression coefficients for reliability on reference population size being β = 0.783 and 0.756, respectively (Figure 2A). As seen in Figure 2A, as the reference population size increased from 100 to 10,000, the average imputation reliabilities of Beagle5.1 increased from 0.75 to 0.87, whereas the average reliabilities of Minimac4 increased from 0.65 to 0.75.

Changes in SNP density in the target population significantly affect the reliability of Beagle5.1 and Minimac4 (*p* < 10^−4^, β = 0.785 and 0.925). When SNP density increased from 1 to 90%, the average imputation reliabilities increased from 0.25 to 0.99 in Beagle5.1 and from 0.24 to 0.95 in Minimac4 (Figure 2B).

The genetic distance between the target population and the reference population had a very significant impact on the reliability for Beagle5.1 (*p* < 10^−4^, β = -0.852), but not for Minimac4 (*p* = 0.43). When the reference population changed from *P*_1_ to *P*_4_, the average imputation reliabilities with Beagle5.1 decreased from 0.80 to 0.69 (Figure 2C). A similar trend was shown in SNP chip data (Supplementary Figure S1; Supplementary Table S1). In addition, imputation reliability showed a trend of first increasing and then slightly decreasing with an increase in MAF, which was more obvious when the reference population was small or genetically distant.

CVs of imputation reliability varied at different levels for the above factors. For Beagle5.1, the CV of reference population size, proportion of target markers/SNP density, and genetic distance were 0.051, 0.320, and 0.021, respectively, while the CV of reference population size and proportion of target markers/SNP density in Minimac4 were 0.051 and 0.340. These indicate that proportion of target markers/SNP density is the most important factor affecting the imputation reliability in both methods.

The imputation reliabilities (Table 5) of Beagle5.1 ranged from 0.21 to 1.00 under different levels of SNP density and reference population size, while the imputation reliabilities of Minimac4 ranged from 0.14 to 0.95. In most cases, the reliabilities of Beagle5.1 were higher than those of Minimac4, except when SNP density was 1% and the reference population size was greater than 5,000. To obtain *r* ^2^ ≥ 0.8 with at least 100 individuals in a reference population, Beagle5.1 required an SNP density of 10%, but Minimac4 required an SNP density of around 30%. Minimac4 could not achieve imputation accuracies of 100%. The performance of Beagle5.1 in reliability was better than that of Minimac4.

**TABLE 5:** Imputation reliability for different levels of imputation reference population size and SNP density.

Software	Reference population size	Proportion of target markers or SNP density/%					
		1	5	10	30	50	90
Beagle5.1	100	0.21	0.56	0.80	0.97	0.99	1.00
	1,000	0.25	0.78	0.94	0.99	1.00	1.00
	3,000	0.26	0.90	0.97	0.99	1.00	1.00
	5,000	0.26	0.94	0.98	1.00	1.00	1.00
	10,000	0.27	0.96	0.99	1.00	1.00	1.00
Minimac4	100	0.14	0.47	0.63	0.82	0.88	0.94
	1,000	0.20	0.58	0.72	0.86	0.90	0.95
	3,000	0.25	0.63	0.74	0.87	0.91	0.95
	5,000	0.28^a^	0.64	0.75	0.87	0.91	0.95
	10,000	0.33^a^	0.67	0.77	0.87	0.91	0.95

a The imputation reliability of Minimac4 is higher than Beagle5.1 only for these two scenarios.

### Factors Affecting Imputation Error Rate

**FIGURE 3:**
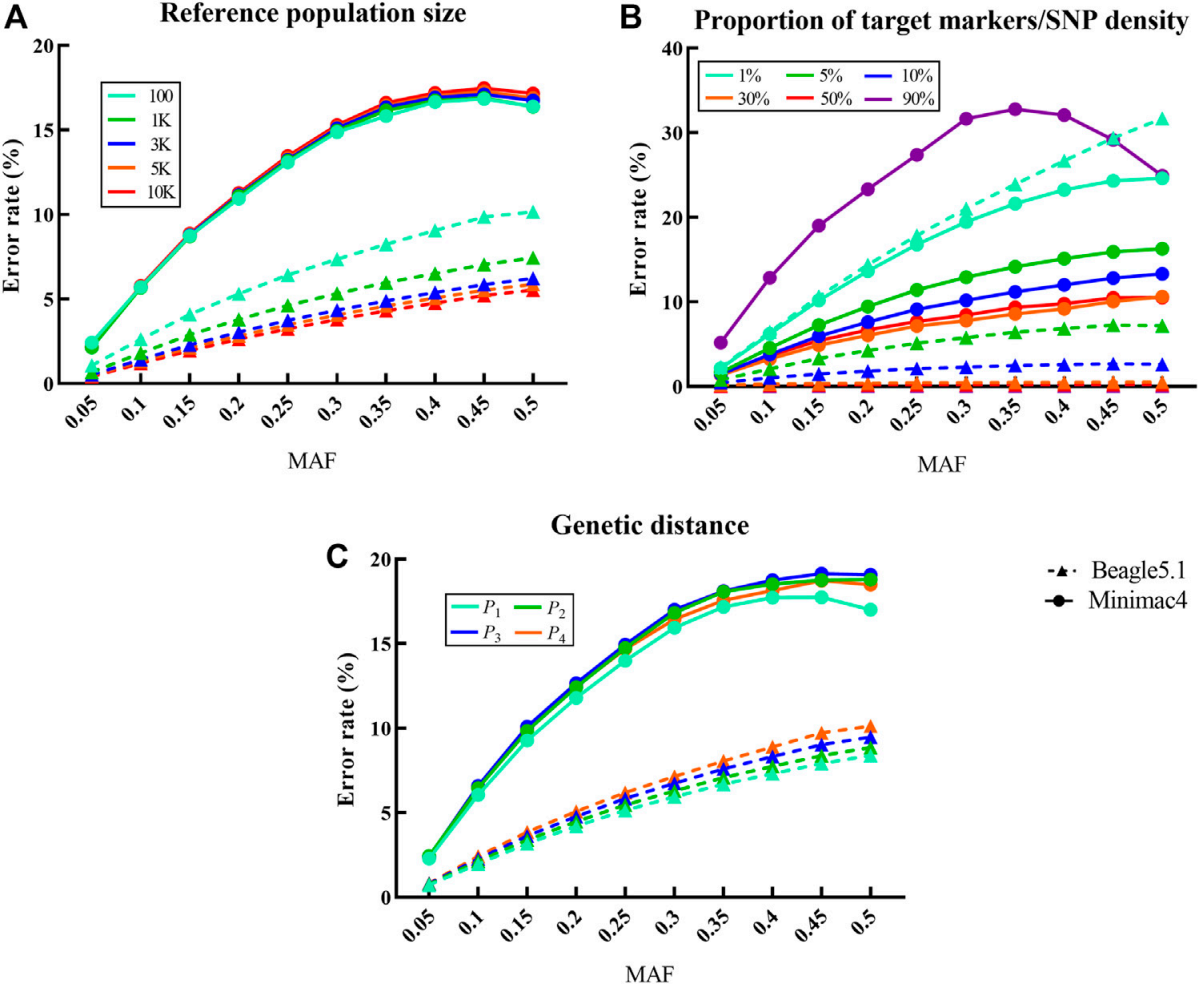
Influence of different factors on the imputation error rate in LCWGS data. For each fixed level of the factors under each scenarios, the average at different levels of all other factors is taken as the error rate. Imputed alleles are binned according to their MAF count in each scenarios. Dotted line with a triangle sign represents Beagle5.1, while the solid line with a round sign represents Minimac4. Different colored signs represent different levels. **(A)** Influence of reference population size on the imputation error rate. **(B)** Influence of proportion of target markers or SNP density on the imputation error rate. **(C)** Influence of genetic distance between reference population and target population on the imputation error rate.

The reference population size (Figure 3) had a very significant effect on the imputation error rate of Beagle5.1 with a negative correlation ( β = -0.431, *p* < 10^−2^), but not with Minimac4. As shown in Figure 3A, when the number of reference samples increased from 100 to 10,000, the average error rates of Beagle5.1 decreased from 6.42 to 3.31%, while the average imputation error rate of Minimac4 hardly changed. As shown in Figure 3B, SNP density has a very significant impact on the imputation error rate in both Beagle5.1 and Minimac4 (*p* < 10^−4^, β = -0.687 and −0.530), and the error rate declined with the increase in SNP density. When the SNP density increased from 1 to 90%, the error rates in Beagle5.1 decreased from 18.43 to 0.07%; the error rates in Minimac4 decreased from 16.22 to 7.35%, corresponding to SNP density increasing from 1% to 50%. Although the genetic distance between the target population and the reference panel has no significant effects on the average imputation error rates of Beagle5.1 or Minimac4 (*p* = 0.36 and *p* = 0.74), it was observed that the lowest average error rates were 4.61 and 9.97% only when the reference population was *P*_1_ (Figure 3C), and similar results are seen when imputing chip data (Supplementary Figure S2; Supplementary Table S2). In addition, the influence of MAF on the imputation error rate was significant and positively correlated in both methods (*p* ≤ 0.04, 0.268< β < 0.975). But when the conditions are conducive to imputation (such as a larger reference population, higher SNP density, or a closer genetic distance between populations), this effect will be less pronounced.

In Beagle5.1, the CVs of the imputation error rate for reference population size and SNP density were 0.262 and 1.508, respectively, while the CV of the imputation error rate affected by the SNP density in Minimac4 is 0.339. This indicated that SNP density was the most important factor affecting the error rate in both imputation methods. In addition, the uncontrollable factor MAF also has a considerable impact on the error rate.

As seen in Table 6, the imputation error rate ranges of Beagle5.1 and Minimac4 were 0.02–19.15% and 6.79–17.25%, respectively. Only when the SNP density was at the extreme low of 1% did Minimac4 exhibit its advantage. In order to achieve an imputation error rate <10%, the imputation of Beagle required SNP density over 5% or to appropriately reduce the SNP density when increasing reference population size, while Minimac4 required the SNP density above 10% but was less dependent on the size of the reference panel. When the reference sample size was 100 and SNP density was slightly higher than 10%, the error rate was less than 5% for Beagle5.1 but not for Minimac4. The performance of Beagle5.1 was better than that of Minimac4 in most cases in terms of the error rate.

**TABLE 6:** Imputation error rate (%) in the different levels of reference population size and SNP density.

Software	Reference population size	Proportion of target markers or SNP density/%					
		1	5	10	30	50	90
Beagle5.1	100	19.15	11.76	5.77	1.15	0.53	0.16
	1,000	18.44	6.55	1.91	0.43	0.23	0.09
	3,000	18.27	2.99	0.98	0.22	0.12	0.05
	5,000	18.20	2.03	0.68	0.15	0.08	0.04
	10,000	18.10	1.17	0.41	0.09	0.05	0.02
Minimac4	100	17.25^a^	11.41^a^	9.01	6.83	7.09	21.26
	1,000	16.36^a^	10.80	8.64	6.79	7.24	23.31
	3,000	16.00^a^	10.72	8.65	6.88	7.39	24.33
	5,000	15.85^a^	10.70	8.68	6.93	7.46	24.80
	10,000	15.67^a^	10.70	8.74	7.02	7.58	25.46

a The imputation error rate of Minimac4 is lower than Beagle5.1 only for these six scenarios.

### Imputation Runtime

The runtimes to impute to the sequence level taken by the two methods in the 1,000-target sample under all scenarios are summarized in Table 7. As seen in Table 7, both the reference population size and SNP density affected the imputation times. Minimac4 was always faster than Beagle5.1. Reference population size and SNP density hardly affected the imputation times taken for Beagle5.1 (Supplementary Table S3). The imputation time of Beagle5.1 only increased with an increase in the proportion of target markers. Beagle5.1 was only faster than Minimac4 when the percentage of target markers was 1% and the reference population sample was more than 1,000 individuals or when the proportion of target markers was 5% and the reference population sample was 10,000. However, considering the trend that the size of the reference population has little effect on time consumed in Beagle5.1, it is likely that Beagle5.1 will eventually be faster than Minimac4 as the reference population size continues to increase.

**TABLE 7:** Runtime (min) to impute 10 Mb low-coverage whole-genome sequencing data with regard to software, reference population size, and proportion of target markers/SNP density.

Software	Reference population size	Proportion of target markers or SNP density/%					
		1	5	10	30	50	90
Beagle5.1	100	107.28	114.60	114.52	110.37	106.45	103.09
	1,000	108.88	120.42	122.82	121.04	105.25	104.76
	3,000	106.18	119.28	116.05	116.43	102.54	100.29
	5,000	106.48	122.42	122.37	118.86	112.88	103.36
	10,000	110.80	123.02	122.37	120.22	112.03	106.18
Minimac4	100	5.22	7.31	7.00	6.75	5.45	4.59
	1,000	8.37	10.06	9.76	9.25	9.36	9.41
	3,000	11.39	13.47	13.91	14.29	15.52	16.5
	5,000	15.20	17.40	17.11	19.15	21.25	24.95
	10,000	21.23	24.63	24.95	29.55	33.40	35.43

### Comparison of Imputation Accuracies of LCWGS and Chip Array Data

We have calculated the CV of the two imputation accuracy standards in all scenarios. The CV is defined as the ratio of the standard deviation to the mean, and it can indicate the extent of the impact of factors considered on the imputation accuracy. Each row in Table 8 represents a different imputation scenario with the asterisked ones being the most important factor affecting imputation in each situation. It can be seen that the SNP density (the proportion of target markers) was the most important in most scenarios. Compared to Minimac4, the imputation accuracies of Beagle5.1 were affected by more factors under the same condition.

**TABLE 8:** Coefficient of variation of imputation reliability and imputation error rates.

Software	Accuracy criterion	Data type	Coefficient of variation		
			Proportion of target markers / SNP density	Reference population size	Genetic distance
Beagle5.1	Reliability	SNP chip	0.164^a^	0.083	0.051
		LCWG sequencing	0.320^a^	0.051	0.021
	Error rate	SNP chip	0.393	0.541^a^	---
		LCWG sequencing	1.508^a^	0.262	---
Minimac4	Reliability	SNP chip	0.313^a^	0.056	---
		LCWG sequencing	0.340^a^	0.051	---
	Error rate	SNP chip	0.490^a^	---	---
		LCWG sequencing	0.339^a^	---	---

a The most important factor affecting the imputation in each scenario.

A dash (---) indicates that the factor has no significant effect on imputation accuracy in this scenario.

**FIGURE 4:**
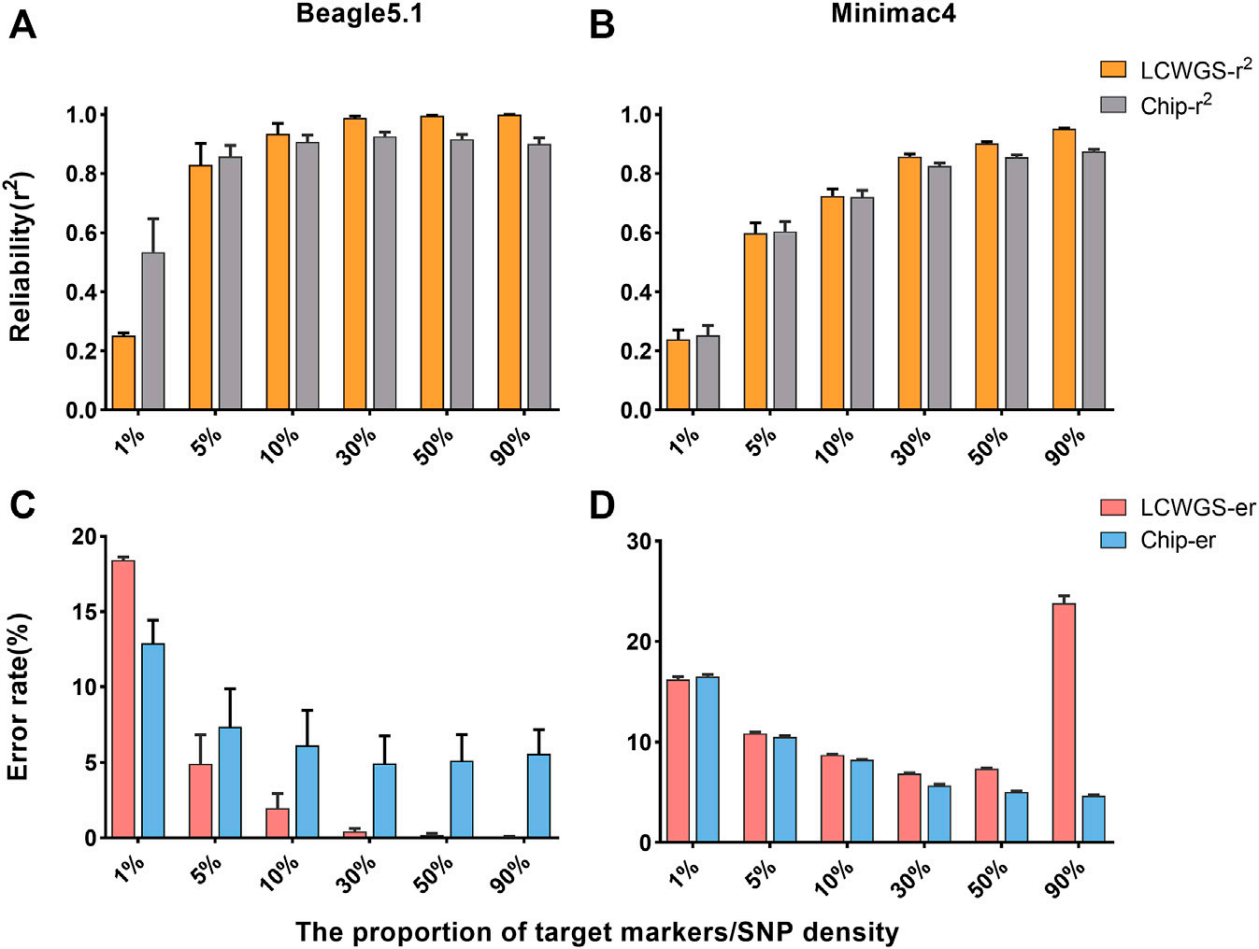
Comparison of imputation accuracy using two types of data. **(A)** Comparison of reliability in Beagle5.1. **(B)** Comparison of reliability in Minimac4. **(C)** Comparison of the error rate in Beagle5.1. **(D)** Comparison of the error rate in Minimac4. LCWGS-r^2^, imputation reliability use LCWG sequence data; Chip-r^2^, imputation reliability use chip data; LCWGS-er, imputation error rate use LCWG sequence data; Chip-er, imputation error rate use chip data.

Although the changes of various factors in this study have almost the same influence on imputation of either LCWGS or chip data, when the level of each factor is the same, there is a difference in imputation between chip data and LCWGS data. Therefore, we directly compared the imputation of the two methods based on the two types of data. The imputation reliability of the two types of data in Beagle5.1 is shown in Figure 4A. When the proportion of target markers is 1%, the average imputation reliability using chip data is 0.51, which is higher than the 0.25 using sequencing data. When the proportion of target markers is greater than 5% (the reliability of the two data types is equal to 0.83), the imputation reliability of LCWGS data completely surpasses that of chip data, and when the proportion of target makers is 30%, the average reliability using LCWGS data can reach the extremely high level of 0.99. Using the Minimac4 method, the reliability with chip data is not less than that with LCWGS data except when the proportion of target markers is 1% and both are 0.24. At other levels, higher imputation reliability can be obtained with LCWGS data (Figure 4B).

Figure 4C shows the error rate of imputation with two types of data in Beagle5.1. When the target marker proportion is ≥5%, the error rate with LCWGS data was lower than that with chip data and can reach at best 4.9%. In contrast, when imputation was with chip data, the error rate in all cases was higher. In Minimac4 (Figure 4D), there was no significant difference in the error rate between imputation with the two types of data, but neither reached the best achieved by Beagle5.1. These showed that in most cases, compared to imputation with chip data, imputation with LCWGS data can achieve higher accuracy imputation, especially in terms of the imputation error rate.

## Discussion

In previous studies (van Binsbergen et al., 2014; Kreiner-Møller et al., 2015; Schurz et al., 2019), the imputation reliability and the imputation error rate were used to assess imputation accuracy. Imputation reliability appears to be a more useful measure with respect to genomic prediction because the nature of imputation reliability coincides with the definition of reliability used for breeding values, and it does not depend on minor allele frequency (MAF). The imputation error rate depends on MAF, which makes it difficult to select the imputed SNPs used for subsequent predictions (Calus et al., 2014).

Imputation accuracy is more problematic for rare variants. Rare variants mean that the locus is almost mono-allelic. The correlation is not defined when one or other of the vectors of true and imputed variants are mono-allelic. Many rare variants will be excluded in subsequent analyses (Pook et al., 2020). Therefore, both imputation reliability and error rate were used to evaluate the accuracy of imputation in this study to consider different applications of the imputed data.

With the development of sequencing technology and the reduction of sequencing costs, choosing SNP chip or LCWGS data has become blurred. In this study, the imputation accuracies of two types of genomic data were different, but under the same scenario, these two types of genetic data have similar influences on significance for each factor considered. That is, the imputation process was not affected by the data type to impute. In the case of the SNP density or proportion of target markers being ≥5%, the imputation performance of Beagle5.1 for the LCWGS data was better than that for the SNP array data, especially in terms of the error rate. This was consistent with findings by Rubinacci et al. (2021), who reported that the reliability of imputation of human sequencing data was the highest in ultrahigh-density chip data, sequencing data, and chip data. Moreover, VanRaden et al. (2015) compared the imputation of low- or medium-density chip data with low-coverage sequencing data with similar costs and found that 1× and 2× deep sequencing data performed better than 10 and 60 K chip data in terms of the imputation error rate and reliability. All these results suggest that low-coverage whole-genome sequencing data has great potential for imputing to whole-genome sequencing resolution. It should be noted that in the case of the proportion of target marker/SNP density being ≤1%, the imputation accuracy of Minimac4 for LCWGS was better than that of Beagle5.1. This might be because SNP markers evenly distributed in the genome can capture more genetic information than LWGS data with a limited number of genetic variants.

Apart from the choice of imputation reference panel, the software used affects the imputation accuracy. In this study, we only compared two software products including Beagle5.1 and Minimac4. Both packages are based on a ‘state-space reduction’ of the hidden Markov models (HMMs) describing haplotype sharing, but the specific simplification methods are different. In Beagle5.1, genotype imputation is based on identity by descent (IBD) and uses the genotypes at the target markers to identify long IBD segments that a target haplotype shares with the reference haplotypes before imputation. It integrates the identified IBD fragments of different lengths into a subset that contains almost the same information as the complete reference haplotypes (Browning et al., 2018). While Minimac4’s model first divides the whole genome into consecutive blocks and iterates only over the unique haplotypes in each genomic block (for imputation with a fixed chromosome length, the length of these blocks is fixed). It uses a reversible mapping function that can reconstruct exactly the state space used by Minimac4 (Das et al., 2016). This will also change the length and number of IBDs in the subset. This is the reason why Beagle5.1 is more sensitive to reference population size. The flexible and computationally intensive method makes Beagle5.1 more suitable for imputing sequencing data in a large reference population size. Under most scenarios, the imputation accuracies of Beagle5.1 were better than those of Minimac4. When the reference population was small, Minimac4 had better performance in the error rate than Beagle5.1. This was consistent with the results of Korkuć et al. (2019). It should be mentioned that when the proportion of target markers was 90%, the imputation error rate of Minimac4 increased abnormally. This was due to the over-correction that caused the error rate of some alleles to be greater than 100% during imputation. To further explain this phenomenon, we rerun our script using Minimac4 when proportions of target markers were 70 and 80%. We still found that the results were similar to that of the density of 90%, and the numbers of alleles with over-correction increased with the increase in density (Supplementary Table S4). This may be a bug of Minimac4.

In the present study, increasing the reference population size led to more accurate imputation, which agreed with other studies (Delaneau et al., 2013; García-Ruiz et al., 2015). A larger reference population can provide more reference haplotypes and the target markers can be more easily matched to the haplotypes, making the reliability higher. Our results are similar to the findings of Hozé et al. (2013), that is, changes of reference population size in Beagle5.1 has a significant impact on the error rate. However, Zhang and Druet (2010) reported that compared with the number of SNPs and genetic distance between populations, the size of the reference population had a relatively small effect on the imputation error rate, which is similar to our findings for Minimac4. This also reflects the differences in calculations between the methods.

In order to obtain high reliability and low error rate imputation, in addition to choosing target markers that more easily match reference haplotypes, we can increase the proportion of target markers or SNP density or select individuals closely related to the target population as the reference population. Another factor that affected the imputation error rate was the difference in MAF, which at first sight may be an unexpected indicator for imputation, especially since haplotypes are used for imputation. However, as shown in other studies (Huang et al., 2009; Oliveira Júnior et al., 2017), since the process of imputation first calculated correlation between reference and target haplotypes and then considered the consistency between the haplotypes, when imputing markers with a higher allele frequency can maintain high correlations, if the frequency between the two genotypes were similar, the marker may not be imputed correctly.

In general, SNP density/the proportion of target markers should be considered first. In this study, when the proportion of target markers was less than 1%, the imputation results in all cases were very poor except that the reliability of imputing chip data with Beagle5.1 could be more than 0.5. An alternative method was a two-step method that has been proven to improve imputation reliability which first imputed the target marker with low-density to a medium-density chip or high-density chip data and then further imputed to sequence resolution (Kreiner-Møller et al., 2015; Wang et al., 2015). A large number of high-coverage sequencing individuals as the reference population data will significantly increase the cost. When the total sequencing depth is fixed (e.g., constrained by budget), balancing the number and depth of sequencing individuals can effectively improve the imputation accuracy, such as using 1,000 individuals with depths of 8× as a reference population have higher imputation reliability than a reference population composed of 500 individuals with 16× (VanRaden et al., 2015). On the other hand, the development and progress of the network database and cloud server technologies also provide opportunities for solving this issue (Das et al., 2018). For instance, the 1,000 Genomes Project and Haplotype Reference Consortium (HRC) public dataset in human research greatly facilitates the application of genotype imputation (Rubinacci et al., 2021). However, in the animal domain, except for the 1,000 Bull Genomes Project (Hayes and Daetwyler, 2019), data sharing channels are still very limited. The use of multiple populations to form a mixed reference population can effectively reduce genetic distance and improve imputation accuracy (Schurz et al., 2019).

## Conclusion

In this study, we have comprehensively analyzed the influence of several factors on the accuracy of genotype imputation. The proportion of target marker/SNP density has a very significant impact on the imputation reliability and the error rate under all imputation situations, which indicate that it is the most important factor in genotype imputation. The imputation performance of Beagle5.1 was better than Minimac4 in most cases, but when the reference population was small, SNP density was low, or genetic distance was large; the imputation accuracy of Beagle5.1 was more easily affected than that of Minimac4. Compared with Minimac4, Beagle5.1 can achieve better imputation performance with relatively relaxed conditions, which was more obvious when the LCWG sequencing data was used to impute to sequence data. Except in the case of extremely low SNP density, the imputation accuracy based on sequencing data is usually better than that based on chip data. Our results provided a reference for the application of genotype imputation in domestic animals.

## Data Availability

The raw data supporting the conclusion of this article will be made available by the authors, without undue reservation.
