# Supplementary material for: Comparison of Genotype Imputation for SNP Array and Low-Coverage Whole-Genome Sequencing Data
Source: Front Genet. 2022 Jan 3;12:704118. doi: 10.3389/fgene.2021.704118 (PMC8762119; doi:10.3389/fgene.2021.704118)
Supplement: Supplementary file 1 [file Table1.DOCX]

**Supplementary Text 1.** *msprime* script for simulating

# usage: python3 sim_pig.py > vcf.file

import msprime, sys

from math import log

from math import exp

nhtps = 22000 # number of haplotypes ,so the number of individules is nhtps/2 = 11000

nbp = 10000000 # length of haplotype

mu = 1e-7 # mutation rate per bp

rcn = 1e-7 # recombination rate per bp

genlen = 1 # years per generation

Tori = 58000 # 58000/genlen initial population size, and reference effective size

Nori = 8000 # initial effective population size

Tot01 = 9000 # number of generations back to first differentiation to P0 and P1

# population history of 9000 to 3000 generation

N0 = 10873 # size of P0 population

N11 = 6400 # initial effective population size of P1 population

m10 = 2.1e-5 #migration rate between P0 and P1, m01 in manuscript

T1t4 = 3000 #number of generations back to P1-P4 split, T1 in manuscript

N1 = 1600 # effective population size of P1 until split

N4 = 1400 # initial effective population size of P4 population

m40 = 3.2e-5 # migration rate between P0 and P4

m14 = 3.7e-4 # migration rate between P1 and P4

r0 = 0.0009 # growth rates per generation of p0

r14 = 0.002 # growth rates per generation of P1 and P4 in 3000-200 generation

T14 = 200 # number of generations back to P2 out of P1, T2 in manuscript

r1 = 0.009 # accelerated growth rate of P1 because breeding

r4 =0.0078

N2 = 1000 # effective population size of P2 population

m12 = 1.1e-3 # migration rate between P1 and P2

Trec = 20 # number of generations back to P3 population generated by

# crossbreeding,T4 in manuscript

N3 = 1200 # population size of P3

r23 = 0.22 # growth rate in most recent generations of P2 and P3

r41 = 0.021

m13 = 6.7e-4

m23 = 5.2e-3 # migration rate between P2 and P3

m34 = 1.6e-3 # migration rate between P3 and P4

# Np0 is P0, Np1 is P1……

Np0 = 0

Np1 = nhtps

Np2 = 6000

Np3 = 6000

Np4 = 6000

pop_config = [

msprime.PopulationConfiguration(sample_size=Np0, initial_size=N0*exp(r0*T1t4), growth_rate=r0),

msprime.PopulationConfiguration(sample_size=Np1, initial_size=N11*exp(r14*(T1t4-T14))*exp(r1*(T14-Trec))*exp(r41*Trec), growth_rate=r41),

msprime.PopulationConfiguration(sample_size=Np2, initial_size=N2*exp(r23*Trec), growth_rate=r23),

msprime.PopulationConfiguration(sample_size=Np3, initial_size=N3*exp(r23*Trec), growth_rate=r23),

msprime.PopulationConfiguration(sample_size=Np4, initial_size=N4*exp(r14*(T1t4-T14))*exp(r4*(T14-Trec))*exp(r41*Trec), growth_rate=r41)

]

mig_matrix = [

[0,m10,0,0,m40],

[m10,0,m12,m13,m14],

[0,m12,0,m23,0],

[0,m13,m23,0,m34],

[m40,m14,0,m34,0]

]

# recent change in growth rate

rec_event = [

msprime.MigrationRateChange(time=Trec, rate=0,matrix_index=(1,2)),

msprime.MigrationRateChange(time=Trec, rate=0,matrix_index=(1,3)),

msprime.MigrationRateChange(time=Trec, rate=0,matrix_index=(2,1)),

msprime.MigrationRateChange(time=Trec, rate=0,matrix_index=(2,3)),

msprime.MigrationRateChange(time=Trec, rate=0,matrix_index=(3,1)),

msprime.MigrationRateChange(time=Trec, rate=0,matrix_index=(3,2)),

msprime.MigrationRateChange(time=Trec, rate=0,matrix_index=(3,4)),

msprime.MigrationRateChange(time=Trec, rate=0,matrix_index=(4,3)),

msprime.PopulationParametersChange(time=Trec, growth_rate=0, population_id=2),

msprime.PopulationParametersChange(time=Trec, growth_rate=0, population_id=3),

msprime.PopulationParametersChange(time=Trec, growth_rate=r1, population_id=1),

msprime.PopulationParametersChange(time=Trec, growth_rate=r4, population_id=4),

msprime.MassMigration(time=Trec+0.0001, source=2, destination=1, proportion=1.0),

msprime.MassMigration(time=Trec+0.0001, source=3, destination=1, proportion=0.5),

msprime.MassMigration(time=Trec+0.0002, source=3, destination=4, proportion=1.0)

]

# New breed animal populations (P2) are generated due to the advent of breeding

acc_event = [

msprime.PopulationParametersChange(time=T14, growth_rate=r14, population_id=1),

msprime.PopulationParametersChange(time=T14, growth_rate=r14, population_id=4)

]

#P1 and P4 merge, migration changes, population size changes

oto_event = [

msprime.MigrationRateChange(time=T1t4, rate=0),

msprime.PopulationParametersChange(time=T1t4, growth_rate=0, population_id=4),

msprime.MassMigration(time=T1t4, source=4, destination=1, proportion=1.0),

msprime.PopulationParametersChange(time=T1t4, growth_rate=0, population_id=0),

msprime.PopulationParametersChange(time=T1t4+0.0001, initial_size=N1, growth_rate=0, population_id=1),

msprime.MigrationRateChange(time=T1t4+0.0001, rate=m10,matrix_index=(0,1)),

msprime.MigrationRateChange(time=T1t4+0.0001, rate=m10,matrix_index=(1,0))

]

#Out of P0 event (looking back, P1 and P0 merge)

otdw_event = [

msprime.MigrationRateChange(time=Tot01, rate=0),

msprime.MassMigration(time=Tot01+0.0001, source=1, destination=0, proportion=1.0)

]

#initial population size

ori_event = [

msprime.PopulationParametersChange(time=Tori, initial_size=Nori, population_id=0)

]

#cat all the events together

events = rec_event + acc_event + oto_event + otdw_event + ori_event

# run the simulation

treeseq = msprime.simulate(population_configurations=pop_config, migration_matrix=mig_matrix,

demographic_events=events, length=nbp, recombination_rate=rcn, mutation_rate=mu, random_seed=seed)

# print results

with sys.stdout as vcffile:

treeseq.write_vcf(vcffile,2)
