# Supplementary figures and images for: Comparison of Genotype Imputation for SNP Array and Low-Coverage Whole-Genome Sequencing Data
Source: Front Genet. 2022 Jan 3;12:704118. doi: 10.3389/fgene.2021.704118 (PMC8762119; doi:10.3389/fgene.2021.704118)

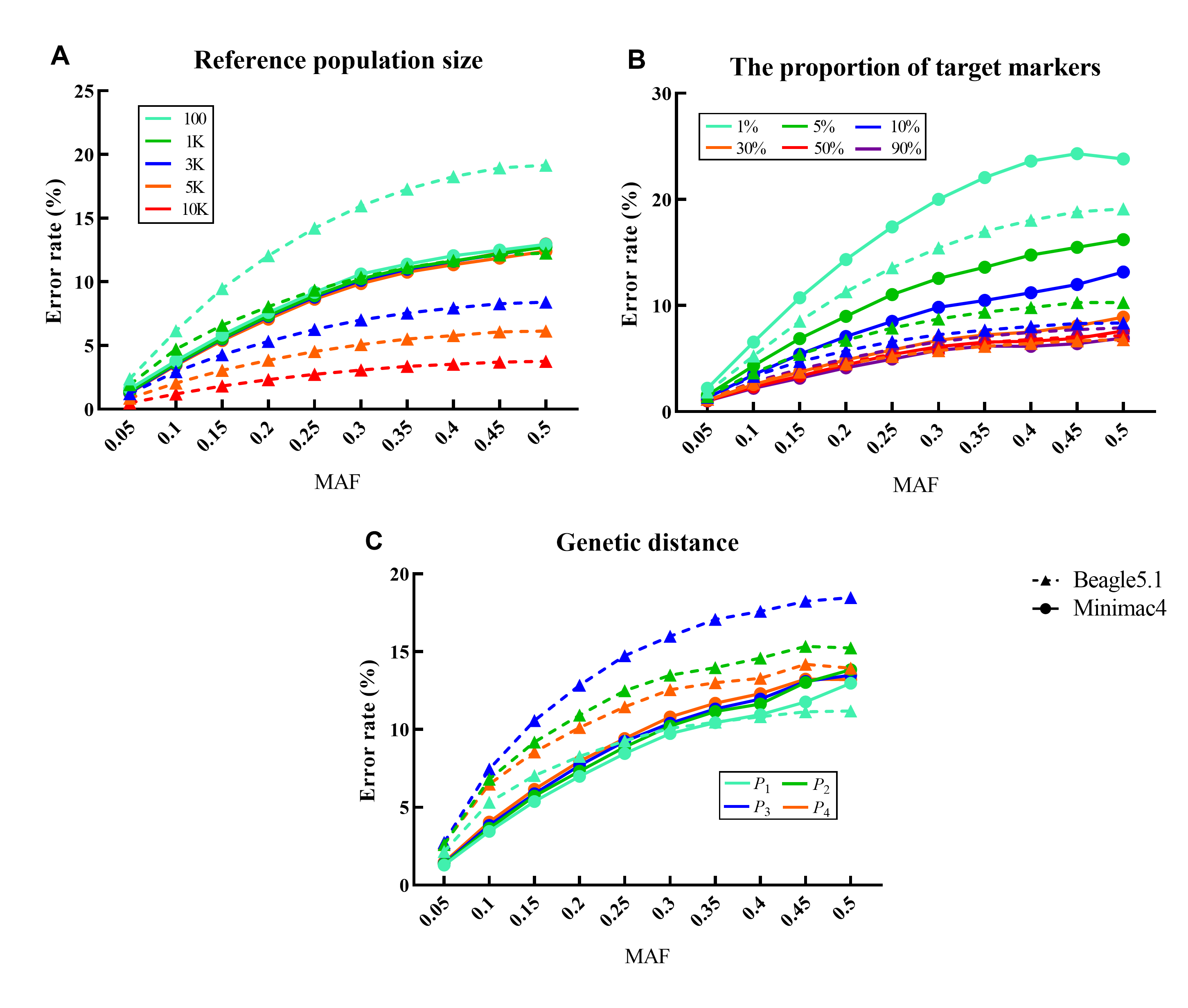

Supplement: Supplementary file 2 [file Image2.TIF]

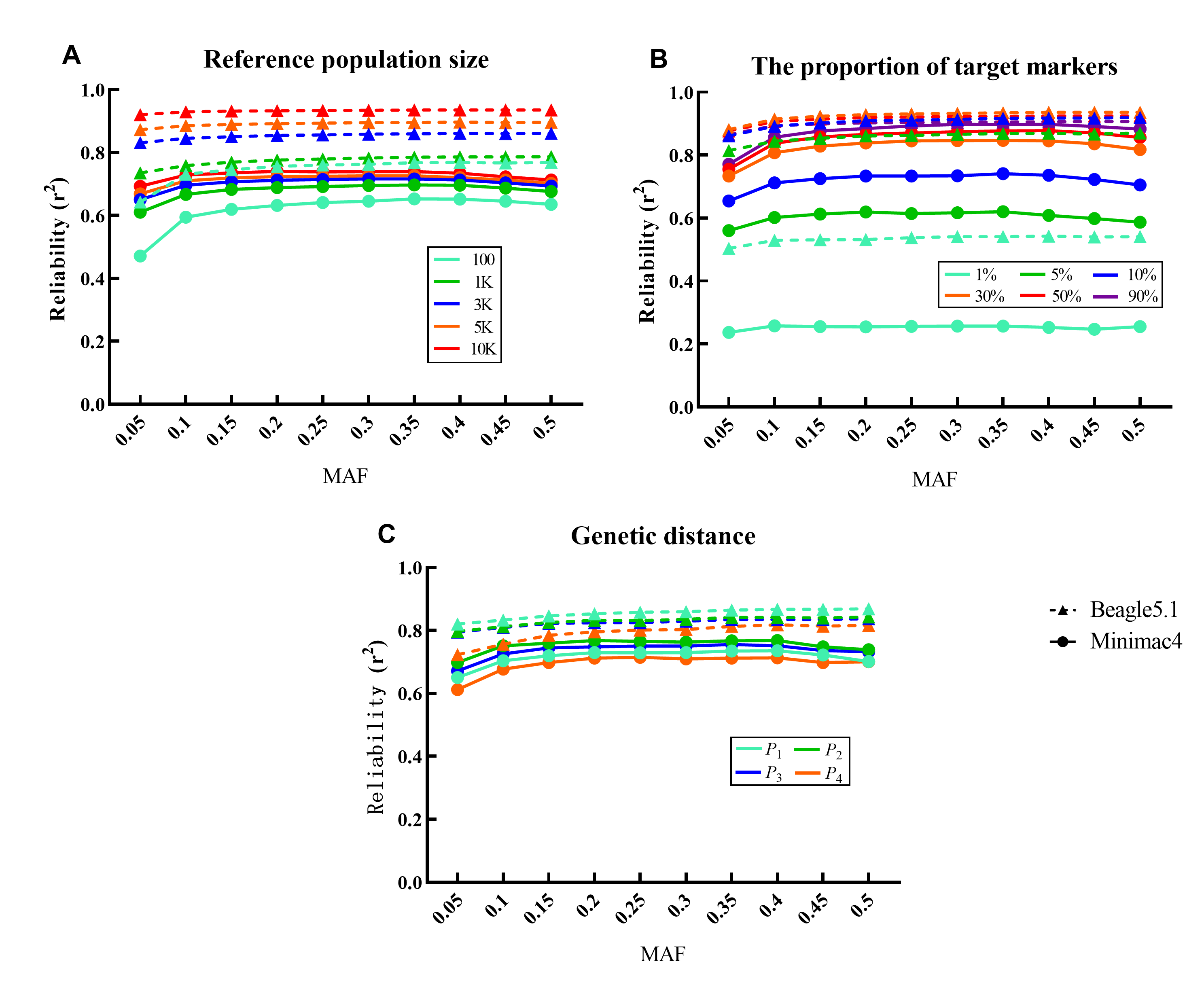

Supplement: Supplementary file 3 [file Image1.TIF]
