# Supplementary material for: Comparison of Genotype Imputation for SNP Array and Low-Coverage Whole-Genome Sequencing Data
Source: Front Genet. 2022 Jan 3;12:704118. doi: 10.3389/fgene.2021.704118 (PMC8762119; doi:10.3389/fgene.2021.704118)
Supplement: Supplementary file 4 [file Table2.DOCX]

Supplementary Material

# Supplementary Figures and Tables

For more information on Supplementary Material and for details on the different file types accepted, please see [here](http://home.frontiersin.org/about/author-guidelines#SupplementaryMaterial). Figures, tables, and images will be published under a Creative Commons CC-BY licence and permission must be obtained for use of copyrighted material from other sources (including re-published/adapted/modified/partial figures and images from the internet). It is the responsibility of the authors to acquire the licenses, to follow any citation instructions requested by third-party rights holders, and cover any supplementary charges.

## Supplementary Figures


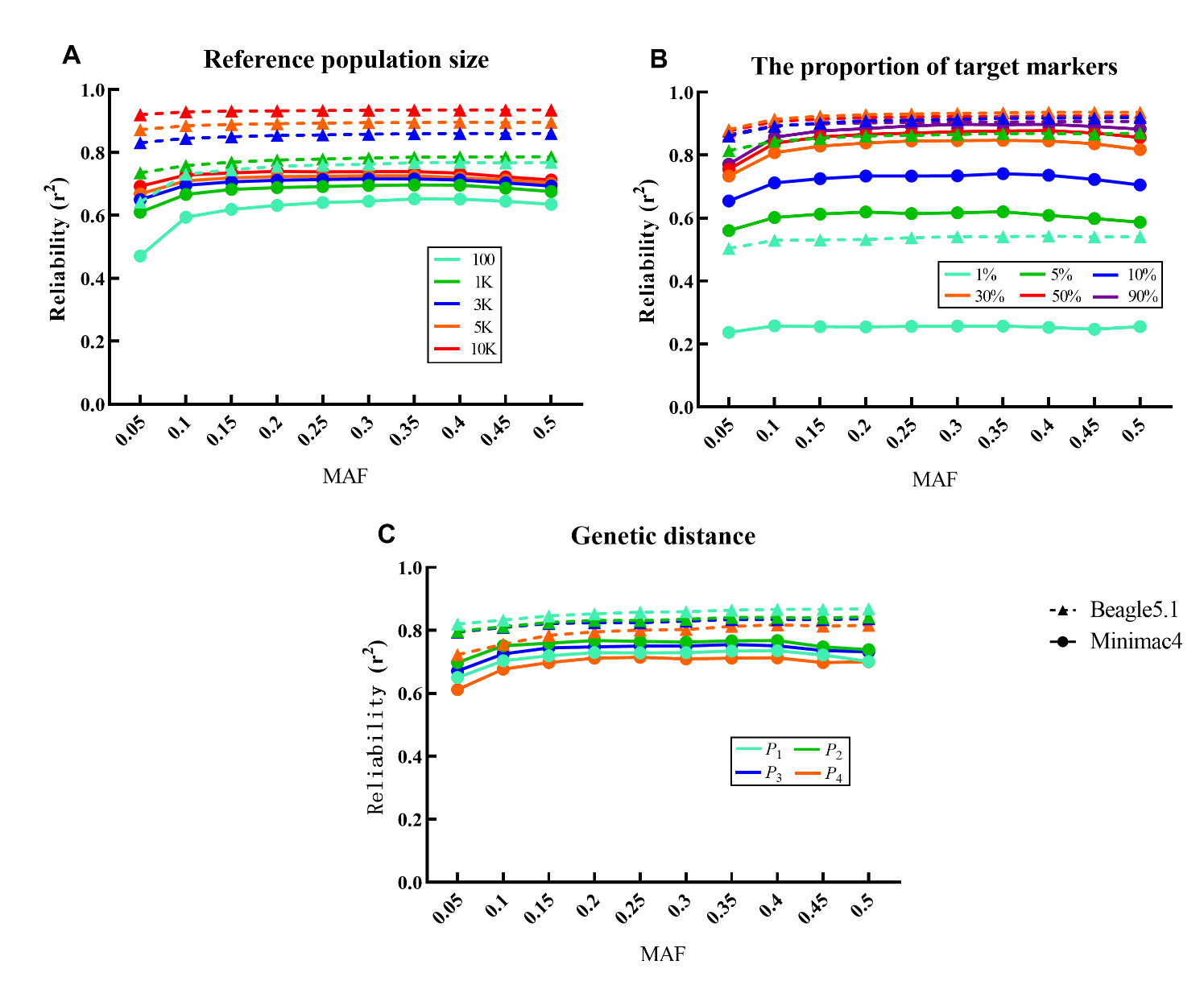


**Supplementary Figure 1.** The influence of different factors on imputation reliability in chip data. For each fixed level of the factors under each scenarios, the average at different levels of all other factors is taken as the reliability. Imputed alleles are binned according to their minor allele count in each scenarios. Dotted line with a triangle sign represents Beagle5.1, while the solid line with a round sign represents Minimac4. Different colored signs represent different levels. (A). Influence of reference population size on imputation reliability; (B). Influence of the proportion of target markers on imputation reliability; (C). The influence of genetic distance between reference population and target population on imputation reliability.


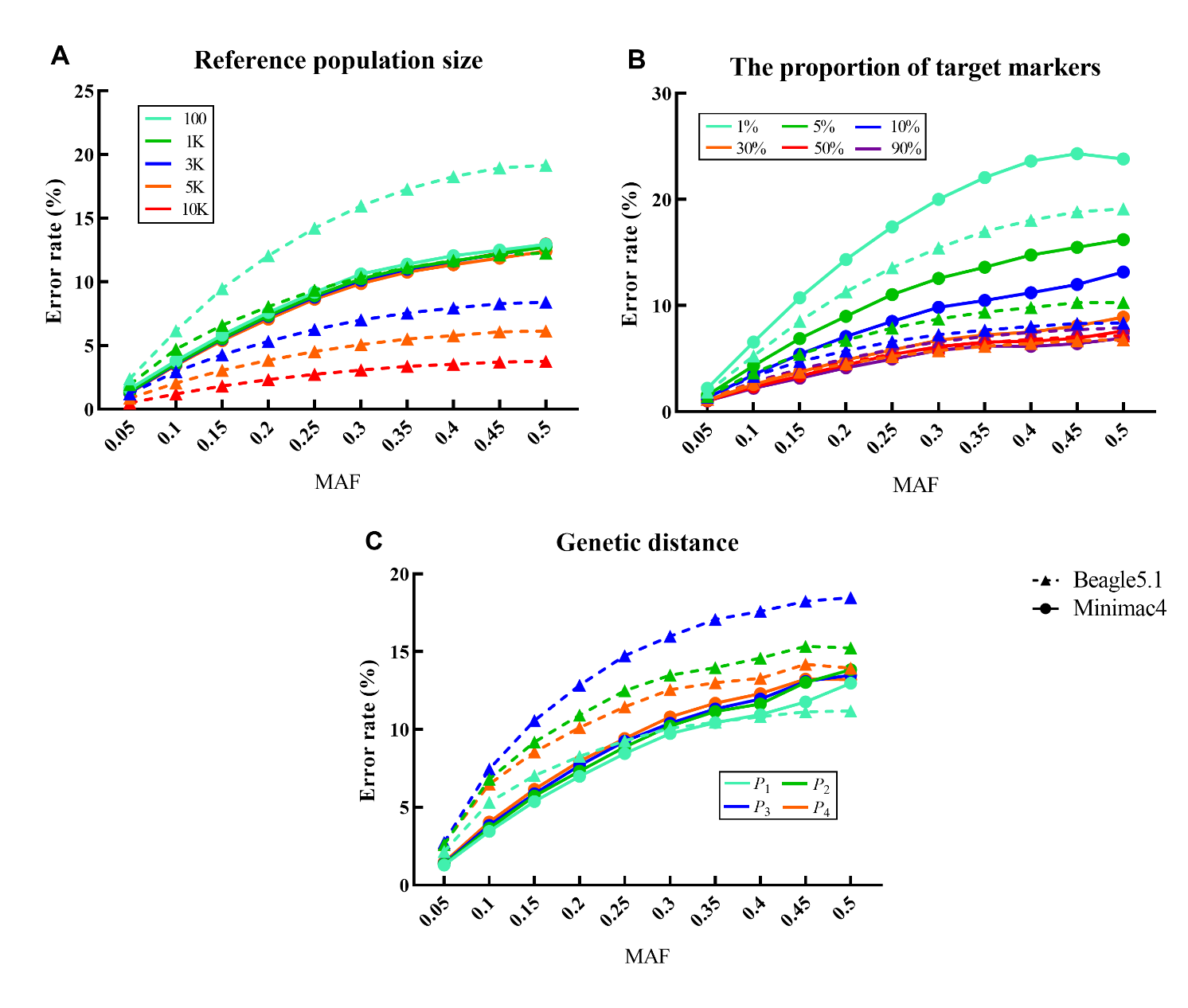


**Supplementary Figure 2.** The influence of different factors on imputation error rate in chip data. For each fixed level of the factors under each scenarios, the average at different levels of all other factors is taken as the error rate. Imputed alleles are binned according to their minor allele count in each scenarios. Dotted line with a triangle sign represents Beagle5.1, while the solid line with a round sign represents Minimac4. Different colored signs represent different levels. **(A).** The influence of reference population size on imputation error rate; **(B).** The influence of proportion of target markers on imputation error rate; **(C).** The influence of genetic distance between reference population and target population on imputation error rate.

## Supplementary Tables

**Supplementary Table 1.** Summary of imputation reliability by reference population size and proportion of target markers

| Software | Reference population size | proportion of target markers/% | | | | | |
| --- | --- | --- | --- | --- | --- | --- | --- |
|  |  | 1 | 5 | 10 | 30 | 50 | 90 |
| Beagle5.1 | 100 | 0.15 | 0.75 | 0.85 | 0.91 | 0.92 | 0.91 |
|  | 1 000 | 0.43 | 0.79 | 0.85 | 0.88 | 0.86 | 0.86 |
|  | 3 000 | 0.61 | 0.88 | 0.92 | 0.92 | 0.91 | 0.88 |
|  | 5 000 | 0.69 | 0.92 | 0.94 | 0.94 | 0.93 | 0.92 |
|  | 10 000 | 0.79 | 0.95 | 0.97 | 0.97 | 0.96 | 0.96 |
| Minimac4 | 100 | 0.15 | 0.48 | 0.63 | 0.78 | 0.82 | 0.85 |
|  | 1 000 | 0.21 | 0.59 | 0.72 | 0.82 | 0.85 | 0.88* |
|  | 3 000 | 0.26 | 0.63 | 0.74 | 0.83 | 0.86 | 0.88 |
|  | 5 000 | 0.29 | 0.65 | 0.75 | 0.84 | 0.87 | 0.88 |
|  | 10 000 | 0.34 | 0.67 | 0.76 | 0.84 | 0.87 | 0.89 |

Note: *The imputation reliability of Minimac4 is higher than Beagle5.1.

**Supplementary Table 2.** The summary of imputation error rate (%) by reference populations size and proportion of target markers

| Software | Reference population size | proportion of target markers/% | | | | | |
| --- | --- | --- | --- | --- | --- | --- | --- |
|  |  | 1 | 5 | 10 | 30 | 50 | 90 |
| Beagle5.1 | 100 | 16.74 | 16.44 | 14.02 | 11.54 | 11.09 | 10.49 |
|  | 1 000 | 15.37 | 8.65 | 8.58 | 5.89 | 6.56 | 7.84 |
|  | 3 000 | 13.15 | 5.69 | 4.09 | 3.70 | 4.10 | 4.84 |
|  | 5 000 | 11.19 | 3.92 | 2.67 | 2.31 | 2.59 | 3.09 |
|  | 10 000 | 8.04 | 2.13 | 1.38 | 1.17 | 1.31 | 1.59 |
| Minimac4 | 100 | 17.17 | 10.89* | 8.43* | 5.90* | 5.25* | 4.86* |
|  | 1 000 | 16.69 | 10.47 | 8.15* | 5.72* | 5.07* | 4.67* |
|  | 3 000 | 16.38 | 10.43 | 8.17 | 5.75 | 5.08 | 4.65* |
|  | 5 000 | 16.22 | 10.44 | 8.21 | 5.09 | 4.65 | 4.65 |
|  | 10 000 | 16.06 | 10.47 | 8.27 | 5.81 | 5.12 | 4.65 |

Note: *The imputation error rate of Minimac4 is lower than Beagle5.1.

**Supplementary Table 3.** Time to impute SNP chip data (min) in relation to software, reference population size and proportion of target markers

| Software | | Reference population size | Proportion of target markers/% | | | | | |
| --- | --- | --- | --- | --- | --- | --- | --- | --- |
|  |  |  | 1 | 5 | 10 | 30 | 50 | 90 |
| Beagle5.1 | 100 | | 2.87 | 6.03 | 10.50 | 28.93 | 43.28 | 87.20 |
|  | 1 000 | | 2.93^*^ | 6.32 | 10.63 | 29.46 | 43.52 | 78.28 |
|  | 3 000 | | 2.72^*^ | 5.87 | 10.85 | 28.46 | 42.42 | 78.12 |
|  | 5 000 | | 3.10^*^ | 7.00 | 11.10 | 29.27 | 44.87 | 79.35 |
|  | 10 000 | | 2.63^*^ | 6.13^*^ | 10.43 | 27.45 | 42.48 | 77.55 |
| Minima4 | 100 | | 2.57 | 3.12 | 3.87 | 3.73 | 3.58 | 4.87 |
|  | 1 000 | | 3.20 | 3.77 | 4.60 | 5.80 | 6.08 | 9.35 |
|  | 3 000 | | 4.10 | 4.30 | 5.78 | 8.85 | 12.67 | 16.38 |
|  | 5 000 | | 4.38 | 4.63 | 6.50 | 11.18 | 15.75 | 20.47 |
|  | 10 000 | | 4.87 | 7.02 | 8.28 | 15.45 | 21.83 | 27.83 |

Note: *The imputation time of Beagle5.1 is faster than Minimac4.

**Supplementary Table 4.** Number of alleles with over-correction

| Reference population size | Proportion of target markers or SNP density/% | |
| --- | --- | --- |
|  | 70 | 80 |
| 100 | 425 | 6 025 |
| 1 000 | 647 | 7 467 |
| 3 000 | 718 | 8 052 |
| 5 000 | 774 | 8 333 |
| 10 000 | 827 | 8 624 |

# Supplementary Data

Supplementary Material should be uploaded separately on submission. Please include any supplementary data, figures and/or tables. All supplementary files are deposited to FigShare for permanent storage and receive a DOI.

Supplementary material is not typeset so please ensure that all information is clearly presented, the appropriate caption is included in the file and not in the manuscript, and that the style conforms to the rest of the article. To avoid discrepancies between the published article and the supplementary material, please do not add the title, author list, affiliations or correspondence in the supplementary files.

**Supplementary Text 1.** *msprime* script for simulating

| # usage: python3 sim_pig.py > vcf.file  import msprime, sys  from math import log  from math import exp  nhtps = 22000 # number of haplotypes ,so the number of individules is nhtps/2 = 11000  nbp = 10000000 # length of haplotype  mu = 1e-7 # mutation rate per bp  rcn = 1e-7 # recombination rate per bp  genlen = 1 # years per generation  Tori = 58000 # 58000/genlen initial population size, and reference effective size  Nori = 8000 # initial effective population size  Tot01 = 9000 # number of generations back to first differentiation to P0 and P1  # population history of 9000 to 3000 generation  N0 = 10873 # size of P0 population  N11 = 6400 # initial effective population size of P1 population  m10 = 2.1e-5 #migration rate between P0 and P1, m01 in manuscript  T1t4 = 3000 #number of generations back to P1-P4 split, T1 in manuscript  N1 = 1600 # effective population size of P1 until split  N4 = 1400 # initial effective population size of P4 population  m40 = 3.2e-5 # migration rate between P0 and P4  m14 = 3.7e-4 # migration rate between P1 and P4  r0 = 0.0009 # growth rates per generation of p0  r14 = 0.002 # growth rates per generation of P1 and P4 in 3000-200 generation  T14 = 200 # number of generations back to P2 out of P1, T2 in manuscript  r1 = 0.009 # accelerated growth rate of P1 because breeding  r4 =0.0078  N2 = 1000 # effective population size of P2 population  m12 = 1.1e-3 # migration rate between P1 and P2  Trec = 20 # number of generations back to P3 population generated by  # crossbreeding,T4 in manuscript  N3 = 1200 # population size of P3  r23 = 0.22 # growth rate in most recent generations of P2 and P3  r41 = 0.021  m13 = 6.7e-4  m23 = 5.2e-3 # migration rate between P2 and P3  m34 = 1.6e-3 # migration rate between P3 and P4  # Np0 is P0, Np1 is P1……  Np0 = 0  Np1 = nhtps  Np2 = 6000  Np3 = 6000  Np4 = 6000  pop_config = [  msprime.PopulationConfiguration(sample_size=Np0, initial_size=N0*exp(r0*T1t4), growth_rate=r0),  msprime.PopulationConfiguration(sample_size=Np1, initial_size=N11*exp(r14*(T1t4-T14))*exp(r1*(T14-Trec))*exp(r41*Trec), growth_rate=r41),  msprime.PopulationConfiguration(sample_size=Np2, initial_size=N2*exp(r23*Trec), growth_rate=r23),  msprime.PopulationConfiguration(sample_size=Np3, initial_size=N3*exp(r23*Trec), growth_rate=r23),  msprime.PopulationConfiguration(sample_size=Np4, initial_size=N4*exp(r14*(T1t4-T14))*exp(r4*(T14-Trec))*exp(r41*Trec), growth_rate=r41)  ]  mig_matrix = [  [0,m10,0,0,m40],  [m10,0,m12,m13,m14],  [0,m12,0,m23,0],  [0,m13,m23,0,m34],  [m40,m14,0,m34,0]  ]  # recent change in growth rate  rec_event = [  msprime.MigrationRateChange(time=Trec, rate=0,matrix_index=(1,2)),  msprime.MigrationRateChange(time=Trec, rate=0,matrix_index=(1,3)),  msprime.MigrationRateChange(time=Trec, rate=0,matrix_index=(2,1)),  msprime.MigrationRateChange(time=Trec, rate=0,matrix_index=(2,3)),  msprime.MigrationRateChange(time=Trec, rate=0,matrix_index=(3,1)),  msprime.MigrationRateChange(time=Trec, rate=0,matrix_index=(3,2)),  msprime.MigrationRateChange(time=Trec, rate=0,matrix_index=(3,4)),  msprime.MigrationRateChange(time=Trec, rate=0,matrix_index=(4,3)),  msprime.PopulationParametersChange(time=Trec, growth_rate=0, population_id=2),  msprime.PopulationParametersChange(time=Trec, growth_rate=0, population_id=3),  msprime.PopulationParametersChange(time=Trec, growth_rate=r1, population_id=1),  msprime.PopulationParametersChange(time=Trec, growth_rate=r4, population_id=4),  msprime.MassMigration(time=Trec+0.0001, source=2, destination=1, proportion=1.0),  msprime.MassMigration(time=Trec+0.0001, source=3, destination=1, proportion=0.5),  msprime.MassMigration(time=Trec+0.0002, source=3, destination=4, proportion=1.0)  ]  # New breed animal populations (P2) are generated due to the advent of breeding  acc_event = [  msprime.PopulationParametersChange(time=T14, growth_rate=r14, population_id=1),  msprime.PopulationParametersChange(time=T14, growth_rate=r14, population_id=4)  ]  #P1 and P4 merge, migration changes, population size changes  oto_event = [  msprime.MigrationRateChange(time=T1t4, rate=0),  msprime.PopulationParametersChange(time=T1t4, growth_rate=0, population_id=4),  msprime.MassMigration(time=T1t4, source=4, destination=1, proportion=1.0),  msprime.PopulationParametersChange(time=T1t4, growth_rate=0, population_id=0),  msprime.PopulationParametersChange(time=T1t4+0.0001, initial_size=N1, growth_rate=0, population_id=1),  msprime.MigrationRateChange(time=T1t4+0.0001, rate=m10,matrix_index=(0,1)),  msprime.MigrationRateChange(time=T1t4+0.0001, rate=m10,matrix_index=(1,0))  ]  #Out of P0 event (looking back, P1 and P0 merge)  otdw_event = [  msprime.MigrationRateChange(time=Tot01, rate=0),  msprime.MassMigration(time=Tot01+0.0001, source=1, destination=0, proportion=1.0)  ]  #initial population size  ori_event = [  msprime.PopulationParametersChange(time=Tori, initial_size=Nori, population_id=0)  ]  #cat all the events together  events = rec_event + acc_event + oto_event + otdw_event + ori_event  # run the simulation  treeseq = msprime.simulate(population_configurations=pop_config, migration_matrix=mig_matrix,  demographic_events=events, length=nbp, recombination_rate=rcn, mutation_rate=mu, random_seed=seed)  # print results  with sys.stdout as vcffile:  treeseq.write_vcf(vcffile,2) |
| --- |
